# Fusion Oncogenes in Patients With Locally Advanced or Distant Metastatic Differentiated Thyroid Cancer

**DOI:** 10.1210/clinem/dgad500

**Published:** 2023-08-25

**Authors:** Gaoda Ju, Yuqing Sun, Hao Wang, Xin Zhang, Zhuanzhuan Mu, Di Sun, Lisha Huang, Ruijue Lin, Tao Xing, Wuying Cheng, Jun Liang, Yan-Song Lin

**Affiliations:** Department of Medical Oncology, Key Laboratory of Carcinogenesis & Translational Research (Ministry of Education/Beijing), Peking University Cancer Hospital and Institute, Beijing, 100142, China; Department of Nuclear Medicine, State Key Laboratory of Complex Severe and Rare Diseases, Peking Union Medical College (PUMC) Hospital, Chinese Academy of Medical Sciences & PUMC, Beijing, 100730, China; Beijing Key Laboratory of Molecular Targeted Diagnosis and Therapy in Nuclear Medicine, Beijing, 100730, China; Department of Oncology, Peking University International Hospital, Peking University, Beijing, 102206, China; Department of Nuclear Medicine, State Key Laboratory of Complex Severe and Rare Diseases, Peking Union Medical College (PUMC) Hospital, Chinese Academy of Medical Sciences & PUMC, Beijing, 100730, China; Beijing Key Laboratory of Molecular Targeted Diagnosis and Therapy in Nuclear Medicine, Beijing, 100730, China; Department of Oncology, Qingdao Municipal Hospital, School of Medicine, Qingdao University, Qingdao, 266011, China; Department of Nuclear Medicine, State Key Laboratory of Complex Severe and Rare Diseases, Peking Union Medical College (PUMC) Hospital, Chinese Academy of Medical Sciences & PUMC, Beijing, 100730, China; Beijing Key Laboratory of Molecular Targeted Diagnosis and Therapy in Nuclear Medicine, Beijing, 100730, China; Department of Nuclear Medicine, State Key Laboratory of Complex Severe and Rare Diseases, Peking Union Medical College (PUMC) Hospital, Chinese Academy of Medical Sciences & PUMC, Beijing, 100730, China; Beijing Key Laboratory of Molecular Targeted Diagnosis and Therapy in Nuclear Medicine, Beijing, 100730, China; Department of Nuclear Medicine, State Key Laboratory of Complex Severe and Rare Diseases, Peking Union Medical College (PUMC) Hospital, Chinese Academy of Medical Sciences & PUMC, Beijing, 100730, China; Beijing Key Laboratory of Molecular Targeted Diagnosis and Therapy in Nuclear Medicine, Beijing, 100730, China; Department of Medical, Zhejiang Shaoxing Topgen Biomedical Technology Co., Ltd., Shanghai, 201321, China; Department of Technology, Zhejiang Topgen Clinical Laboratory Co., Ltd., Huzhou, 201914, China; Department of Medical Oncology, Key Laboratory of Carcinogenesis & Translational Research (Ministry of Education/Beijing), Peking University Cancer Hospital and Institute, Beijing, 100142, China; Department of Nuclear Medicine, State Key Laboratory of Complex Severe and Rare Diseases, Peking Union Medical College (PUMC) Hospital, Chinese Academy of Medical Sciences & PUMC, Beijing, 100730, China; Beijing Key Laboratory of Molecular Targeted Diagnosis and Therapy in Nuclear Medicine, Beijing, 100730, China; Department of Medical Oncology, Key Laboratory of Carcinogenesis & Translational Research (Ministry of Education/Beijing), Peking University Cancer Hospital and Institute, Beijing, 100142, China; Department of Oncology, Peking University International Hospital, Peking University, Beijing, 102206, China; Department of Nuclear Medicine, State Key Laboratory of Complex Severe and Rare Diseases, Peking Union Medical College (PUMC) Hospital, Chinese Academy of Medical Sciences & PUMC, Beijing, 100730, China; Beijing Key Laboratory of Molecular Targeted Diagnosis and Therapy in Nuclear Medicine, Beijing, 100730, China

**Keywords:** differentiated thyroid cancer, fusion oncogenes, radioactive iodine therapy, *TERT* mutation, next-generation sequencing

## Abstract

**Context:**

Fusion oncogenes are involved in the underlying pathology of advanced differentiated thyroid cancer (DTC), and even the cause of radioactive iodine (RAI)-refractoriness.

**Objective:**

We aimed to investigation between fusion oncogenes and clinicopathological characteristics involving a large-scale cohort of patients with advanced DTC.

**Methods:**

We collected 278 tumor samples from patients with locally advanced (N1b or T4) or distant metastatic DTC. Targeted next-generation sequencing with a 26-gene ThyroLead panel was performed on these samples.

**Results:**

Fusion oncogenes accounted for 29.86% of the samples (72 rearrangement during transfection (*RET*) fusions, 7 neurotrophic tropomyosin receptor kinase (*NTRK*) fusions, 4 anaplastic lymphoma kinase (*ALK*) fusions) and occurred more frequently in pediatric patients than in their adult counterparts (*P* = .003, OR 2.411, 95% CI 1.329-4.311) in our cohort. DTCs with fusion oncogenes appeared to have a more advanced American Joint Committee on Cancer (AJCC)_N and AJCC_M stage (*P* = .0002, OR 15.47, 95% CI 2.54-160.9, and *P* = .016, OR 2.35, 95% CI 1.18-4.81) than those without. DTCs with fusion oncogenes were associated with pediatric radioactive iodine (RAI) refractoriness compared with those without fusion oncogenes (*P* = .017, OR 4.85, 95% CI 1.29-15.19). However, in adult DTCs, those with fusion oncogenes were less likely to be associated with RAI refractoriness than those without (*P* = .029, OR 0.50, 95% CI 0.27-0.95), owing to a high occurrence of the *TERT* mutation, which was the most prominent genetic risk factor for RAI refractoriness in multivariate logistic regression analysis (*P* < .001, OR 7.36, 95% CI 3.14-17.27).

**Conclusion:**

Fusion oncogenes were more prevalent in pediatric DTCs than in their adult counterparts and were associated with pediatric RAI refractoriness, while in adult DTCs, *TERT* mutation was the dominant genetic contributor to RAI refractoriness rather than fusion oncogenes.

Annual new cases of thyroid cancer in China account for more than one-third of global new cases (1). More than 94% of thyroid cancers are differentiated thyroid cancer (DTC), which mainly includes papillary thyroid cancer (PTC) and follicular thyroid carcinoma. Most patients with thyroid cancer have a good prognosis, with a 5-year survival rate of 84.3% in China and 98.3% in the United States (2-4). However, up to 20% of patients with DTC develop locally advanced disease or distant metastases (5-7) and these patients usually have poorer differentiation status, lower avidity for iodine, and shorter survival than patients with DTC at an early stage (8-11). Studies have suggested that multiple gene variants (BRAFV600E, *TERT*, *RET*, *RAS*, etc.) may participate in this process of dedifferentiation through different signaling pathways by distinct genetic alterations. Among these, fusion oncogenes (*RET/NTRK/ALK* fusions), having a high proportion of genetic alterations of PTC (6-46%) and may be involved in the tumorigenesis, dedifferentiation, and progression of DTC, and thereby should be considered as possible biomarkers and drug targets (12-15).

*RET* fusions occur in 10% to 30% of patients with DTC (16-20). In 1990, RET/PTCs were first identified as chimeric genes in PTC encoded by the *RET* gene, which is composed of 3 domains: an extracellular domain containing 4 cadherin-like regions; a transmembrane domain; and a C-terminal tyrosine kinase domain (21, 22). *RET* fusions have been reported to be involved in the tumorigenesis of thyroid carcinoma through the activation of *RET* and its downstream pathways such as the phosphatidylinositol 3-kinase/protein kinase B (*PI3K/AKT*) signaling pathway, mitogen-activated protein kinases (*MAPK*) signaling pathway, and rat sarcoma/extracellular signal-regulated kinase (*RAS/ERK*) signaling pathway (21, 23-29). To date, different types of *RET/PTC* chimeric genes according to partner genes have been identified, in which *RET/PTC1* (*CCDC6/RET*) and *RET/PTC3*(*NCOA4/RET*) account for >90% of all *RET/PTCs* (30). *NTRK* fusions can be detected in 2% to 3% of sporadic PTC (12, 14, 15), playing an important role in the pathogenesis of thyroid cancer by activating downstream TRK kinase (31). *ALK* fusions occur in 1% to 3% of PTC and are related to tumor dedifferentiation and poor prognosis in PTC (12, 32-35). The proliferation of thyroid cancer cells induced by ALK fusions in vitro and in nude mice was blocked by crizotinib and TAE684 (ALK inhibitors), which indicated ALK fusions as a potential therapeutic target of thyroid carcinomas (32).

Moreover, when it comes to molecular characterization toward age, pediatric DTCs (pDTCs) are distinct from the adult counterparts (aDTCs) (36, 37). Pediatric PTC (pPTC) is characterized by a higher incidence of gene fusions, and a lower frequency of point mutations in proto-oncogenes associated with PTC. Specifically, fusions are more common in pPTC than in adult PTC (aPTC) (50-60% vs 15%) (38). The *CCDC6/RET* fusion is the most common *RET/PTC* variants in children with sporadic PTC, and the *NCOA4/RET* fusion occurs in a high proportion of radiation-induced cases (39). However, the common genetic events in aDTC, such as *BRAF* gene mutation (36.0-83.0%) and *RAS* mutation (10.0-55.0%), are relatively rare in pDTC (0.0%-3.2%, 2.5-13.0%), especially in younger patients (40-45).

Growing evidence has indicated that fusion oncogenes are usually associated with the more advanced stage in DTC, and tumors harboring fusion oncogenes seem to be more prone to develop lymph node metastases and distant metastases than their fusion oncogene-negative counterparts (46-48). The survival rates for patients with locally advanced or metastatic DTC decrease markedly with a 10-year survival rate of 56%, and approximately two-third of these patients eventually progress into radioactive iodine (RAI)-refractory DTC (RAIR-DTC), a life-threatening status of DTC with a 10-year survival rate of less than 10% (5, 11, 49-52). Colombo et al have investigated the association between fusion oncogenes and RAIR-DTC, and have found that fusion genes were more frequent in RAI-avid/disease-persistent DTCs, while *BRAF/TERT* mutations were more prevalent in the RAIR/disease-persistent DTCs (53). It is noteworthy that their study only included 4 patients aged under 25 years, who appear to be less representative among pDTCs. Considering the significant difference in molecular alterations and clinicopathological features between pDTC and aDTC, it is necessary to investigate the significance of fusion oncogenes in DTC progression, and the association between fusion oncogenes and RAI response, particularly RAIR-DTC, across age.

In this study, we aimed to identify fusion oncogenes across age and clarify the possible implication of fusion oncogenes and their association with RAI response in pediatric and adult patients with locally advanced or distant metastatic DTC, respectively.

## Material and Methods

### Patient Population

Our study comprised 278 formalin-fixed paraffin-embedded tumor samples from patients with locally advanced (N1b or T4) or distant metastatic DTC, obtained at the Peking Union Medical College Hospital between 2020 and 2022 (Table S1 (54)). This included 216 samples collected from adult patients and 62 samples collected from pediatric patients under the age of 20 years. Detailed pathological and clinical information of tumor tissue samples was obtained. Patients with RAI refractoriness were defined according to the 2015 American Thyroid Association Management Guidelines for Patients with DTC: (1) locoregional or metastatic lesions that never concentrate RAI; (2) tumor tissue that lost iodine uptake function; (3) RAI concentrated in certain lesions but not others; and (4) metastatic lesions that progressed despite iodine uptake (55). The tumor (T), regional lymph node (N), and distant metastasis (M) stages of each patient were identified according to the American Joint Committee on Cancer (AJCC) staging system (8th edition). This study was approved by the Hospital Ethics Committee of Peking Union Medical College Hospital. The approval number was JS-2600 and all participants provided written informed consent.

### Next-Generation Sequencing

The ThyroLead panel (Topgen, China) covering the exonic region of 18 genes (*HRAS, KRAS, NRAS, CDC73, CDKN1B, DICER1, IDH1, MEN1, MTOR, PIK3CA, PTEN, TP53, TSHR, CTNNB1, GNAS, PAX8, AKT1, EIF1AX*), a 1000-bp region in the promoter region of *TERT*, and both exonic and intronic regions for 7 fusion oncogenes (*BRAF, RET, NTRK1/2/3, ALK,* and *PPARG*), was used in our study. The details of DNA isolation, sequence library preparation, and sequencing were described in a previous study (56). Adapter sequences and low-quality reads (with more than 40% of bases failing Q25; reads <70 bp; and low-complexity reads) were removed by Fastp. The reads were aligned to the reference human genome hg19 with BWA-MEM using default parameters (57). The variant calling process and indel realignment were performed using Sentieon TNseq software. Variation annotation was performed according to databases such as COSMIC, The 1000 Genomes Project, ESP, ExAC, gnomAD, and ClinVar with VEP and Annovar. After quality control, samples with uneven depth of coverage, low deduplicated depth, or aberrant insert sizes were discarded in the next analysis. The sequencing coverage and quality statistics of targeted NGS can be seen elsewhere (Table S2 (54)).

### Data Analyses

All analyses were performed using R 4.0.2, IBM SPSS Statistics 26, and GraphPad Prism 9.0. Descriptive statistics were used to analyze categorical variables using frequencies and proportions of the demographic data. Categorical variables were compared between the 2 groups using the chi-square test or Fisher's exact test. Logistic regression was performed to evaluate the associations of genetic alterations with the risk of RAI refractoriness. The odds ratio (OR), 95% CI, and *P* value are reported in the tables. Data from GSE151179 (53) was downloaded from the Gene Expression Omnibus website and was utilized to compare the expression of Na(+)/I(–) symporter (NIS) between wild type PTC and PTC with *BRAF* mutation or PTC with fusions. The expression of *NIS* was compared between the 2 groups by Student's t test. *P* < .05 was considered to be statistically significant.

## Results

### Patient Demographics and Pathological Subtype

The demographics and pathological subtype of 278 Chinese patients with locally advanced and distant metastatic DTC (age range 4.67-81.36 years; n = 166 females and n = 112 males; n = 62 pediatric patients and n = 216 adults) are summarized in Table 1. Tumors were divided on the basis of their dominant histopathologic features: 261 (93.9%) PTC, 12 (4.3%) follicular thyroid carcinoma, 4 (1.4%) poorly differentiated thyroid cancer, and 1 (0.4%) other DTC (Table 1).

**Table 1.** Comparison of clinical characteristics and RAI refractoriness between the Fusions group and the Non-Fusions group

Characteristics	Total n (%)	Fusions n (%)	Non-Fusions n (%)	*P*
**Sex**				
Female	166 (59.7)	49 (59.0)	117 (60.0)	.881
Male	112 (40.3)	34 (41.0)	78 (40.0)	
**Age group**				
Pediatric (≤20)	62 (22.3)	28 (33.7)	34 (17.4)	.003
Adult (>20)	216 (77.7)	55 (66.3)	161 (82.6)	
**Diagnosis**				
PTC	261 (93.9)	81 (97.6)	180 (92.3)	na*
FTC	12 (4.3)	1 (1.2)	11 (5.6)	
PDTC	4 (1.4)	1 (1.2)	3 (1.5)	
Other	1 (0.4)	0 (0.0)	1 (0.6)	
**AJCC_T*^a^***				
T1	89 (34.5)	27 (34.6)	62 (34.4)	.809
T2	45 (17.4)	16 (20.5)	29 (16.1)	
T3	43 (16.7)	13 (16.7)	30 (16.7)	
T4	81 (31.4)	22 (28.2)	59 (32.8)	
**AJCC_N*^a^***				
N0/N1a	30 (11.6)	1 (1.2)	29 (16.2)	.0002
N1b	230 (88.4)	80 (98.8)	150 (83.8)	
AJCC_M*^a^*				
M0	63 (22.7)	11 (13.4)	52 (26.7)	.016
M1	214 (77.3)	71 (86.6)	143 (73.3)	
**RAI refractoriness**				
Yes	124 (44.6)	31 (37.3)	93 (47.7)	.112
No	154 (55.4)	52 (62.7)	102 (52.3)	

Abbreviations: AJCC, American Joint Committee on Cancer; FTC, follicular thyroid carcinoma; PTC, papillary thyroid cancer; PDTC, poorly differentiated thyroid cancer; RAI, radioactive iodine.

^*a*^AJCC_T stage information of 20 patients, AJCC_N stage information of 18 patients and AJCC_M, stage information of 1 patient were uncertain.

*na means no compare between multiple groups due to 0 value. The total number of patients in each characteristics subgroup was used as denominators to calculate percentages both in Fusions group and Non-Fusions group.

### Identification of Patients With Fusion Oncogenes

**Figure 1.**
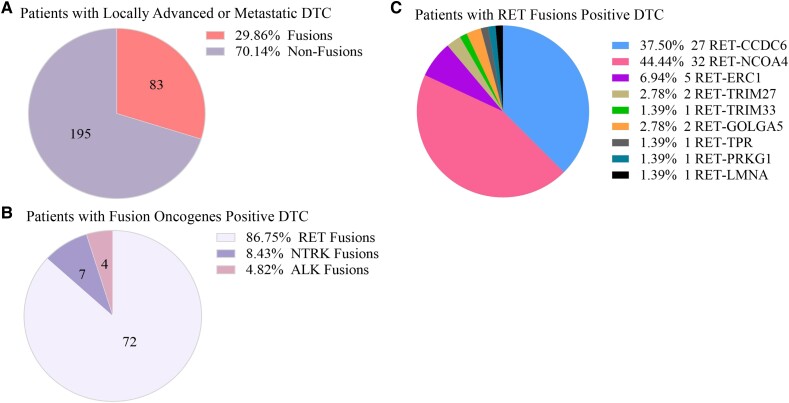
The prevalence of fusion oncogenes in patients with locally advanced or distant metastatic DTC. (A) Fusion oncogenes were detected in 83 of 276 tumors. (B) *RET* fusions were detected in 72 tumors, *NTRK* fusions were detected in 7 tumors, and *ALK* fusions were detected in 4 tumors. (C) Nine types of *RET* fusions were identified in this cohort.

**Figure 2.**
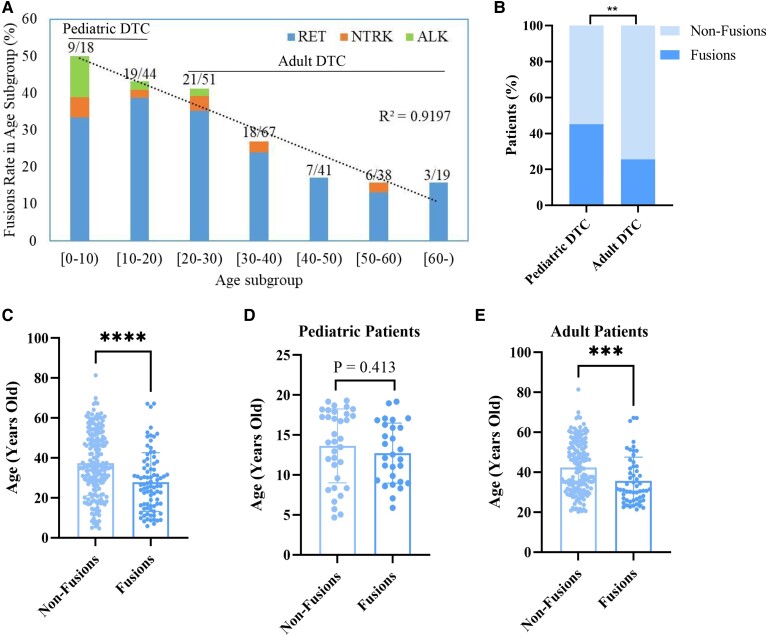
The association of fusion oncogenes with age. (A) The occurrence rate of fusion oncogenes in DTC according to age. (B) The occurrence rate of fusion oncogenes between pediatric and adult patients with DTC. (C) The age of patients between Fusions group and Non-Fusions group. (D) The age of pediatric patients between Fusions group and Non-Fusions group. (E) The age of adult patients between Fusions group and Non-Fusions group.

Of the 278 samples, fusion oncogenes were identified in 83 patients (Fig. 1A), including 72 *RET* fusions (*RET/PTCs*), 7 *NTRK1* fusions, and 4 *ALK-STRN* fusions (Fig. 1B). In this study, 9 different types of *RET/PTCs* were identified, and *RET/PTC3* (*RET-NCOA4*: 44.44%) and *RET/PTC1* (*RET-CCDC6*: 37.50%) were the most common types, accounting for approximately 82.00% of all cases (Fig. 1C). With increasing age, the proportion of patients with fusion oncogenes decreased (Fig. 2A) and the proportion of patients with fusion oncogenes in pediatric patients (<20 years old) was significantly higher than that in adult patients (≥20 years) (45.20% vs 25.50%, *P* < .01) (Fig. 2B). The age of patients with fusion oncogenes (Fusions group) was 27.88 ± 14.71 years old (mean ± SD), which was significantly younger than that of patients without fusion oncogenes (Non-Fusions group) whose age was 37.36 ± 16.13 years (mean ± SD) (*P* < .0001) (Fig. 2C). In addition, patients with *RET-NCOA4* (24.3 ± 12.55 years, mean ± SD) were younger than patients with *RET-CCDC6* (30.95 ± 16.22 years, mean ± SD) (Fig. S1 (54)). Subgroup analysis of the age between the Fusions group and the Non-Fusions group was performed, demonstrating that the age was not significantly different between the Fusions group (12.73 ± 3.76 years old, mean ± SD) and the Non-Fusions group (13.63 ± 4.63 years old, mean ± SD) (*P* = .413) in the pediatric subgroup (Fig. 2D), while the age of the Fusions group (35.60 ± 11.91 years, mean ± SD) was younger than that of the Non-Fusions group (42.37 ± 12.89 years, mean ± SD) (*P* < .001) in the adult subgroup (Fig. 2E).

### Associations of Fusion Oncogenes With Clinicopathological Characteristics and RAI Refractoriness

**Figure 3.**
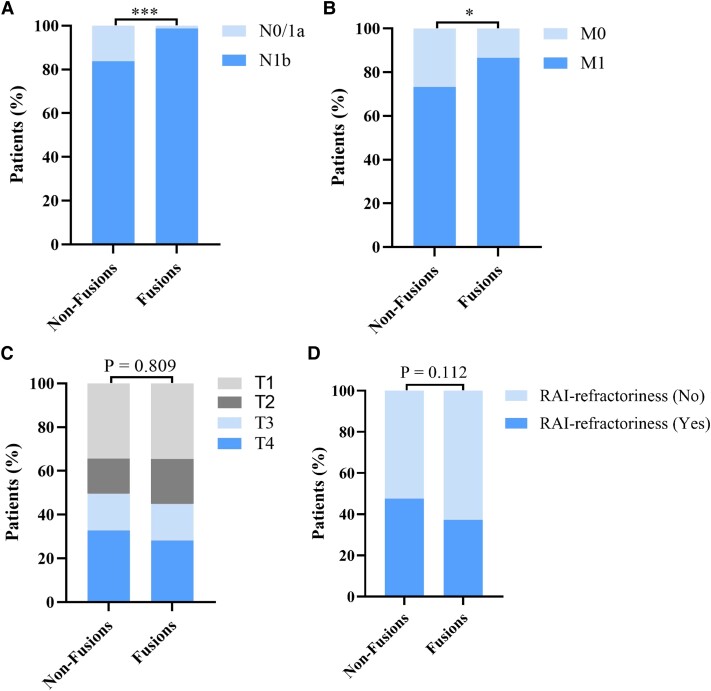
The associations of fusion oncogenes with clinicopathological characteristics and the risk of RAI refractoriness. (A) The association of fusion oncogenes with AJCC_N stage. (B) The association of fusion oncogenes with AJCC_M stage. (C) The association of fusion oncogenes with AJCC_T stage. (D) The association of fusion oncogenes with RAI refractoriness.

**Figure 4.**
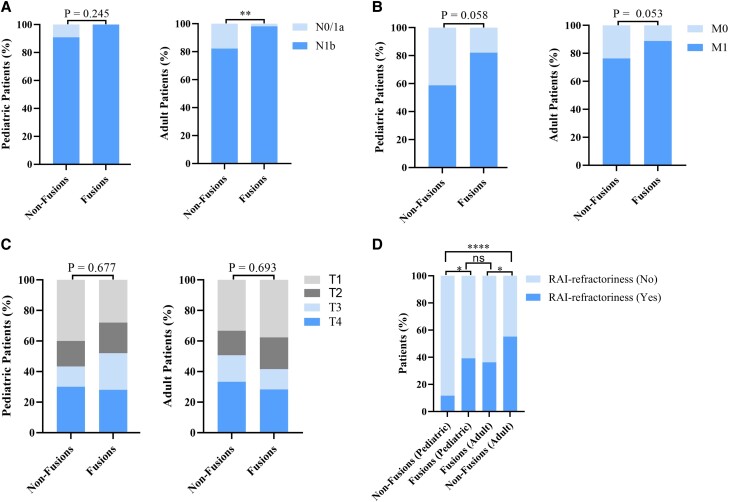
Subgroup analysis of the associations of fusion oncogenes with clinicopathological characteristics and the risk of RAI refractoriness according to age group. (A) The association of fusion oncogenes with AJCC_N stage in pediatric and adult patients, respectively. (B) The association of fusion oncogenes with AJCC_M stage in pediatric and adult patients, respectively. (C) The association of fusion oncogenes with AJCC_T stage in pediatric and adult patients, respectively. (D) The association of fusion oncogenes with RAI refractoriness in pediatric and adult patients, respectively.

On the one hand, we found that DTC with fusion oncogenes was associated with a higher proportion of N1b (*P* = .0002, OR 15.47, 95% CI 2.54-160.9) (Fig. 3A) and M1 (*P* = .016, OR 2.35, 95% CI 1.18-4.81) (Fig. 3B) than DTC without fusion oncogenes. On the other hand, fusion oncogenes did not seem to be statistically associated with the AJCC_T stage (Fig. 3C) or the risk of RAI refractoriness (Fig. 3D). We further evaluated the associations of N stage with the major genetic alterations (mutational ratio >5%) by univariate and multivariate logistic regression analyses. The results (Table S3 (54)) from multivariate logistic regression analysis demonstrated that fusions were the only genetic risk factor for advanced N stage while *RAS* mutation was the only genetic protective factor for advanced N stage. In the next step, logistic regression analysis was performed to estimate the interaction effect of age group and fusion oncogenes on RAI refractoriness. As described in Table 2, both age group and fusion oncogenes were significant contributors to the risk of RAI refractoriness, and the 2-way interaction between age group and fusion oncogenes was significant (*P* = .002, OR 0.103, 95% CI 0.025-0.432). Hence, the associations of fusion oncogenes with clinicopathological characteristics and RAI refractoriness stratified by age group were evaluated. Interestingly, patients in the Fusions group seemed to have a more advanced AJCC_N and AJCC_M stage than patients in the Non-Fusions group, whether in the pediatric or adult subgroup, although not all of the *P* values are less than .05 in Fig. 4A and Fig. 4B. In addition, AJCC_T stage was not significantly associated with fusion oncogenes in either the pediatric or adult subgroup (Fig. 4C). In addition, the associations of fusion oncogenes with RAI refractoriness stratified by age group is shown in Fig. 4D: (1) The proportion of patients with RAI refractoriness is higher in the pediatric Fusions group than that in the pediatric Non-Fusions group (*P* = .012, 39.3% vs 11.7%); (2) the proportion of patients with RAI refractoriness is lower in the adult Fusions group than that in the adult Non-Fusions group (*P* = .029, 36.3% vs 55.2%); (3) the proportion of RAI refractoriness is significantly higher in the adult Non-Fusions group than that in the pediatric Non-Fusions group (*P* < .0001, 55.2% vs 11.7%); (4) no significant difference of RAI refractoriness is observed between the pediatric Fusions group and the adult Fusions group (*P* = .922, 39.3% vs 36.3%). Among pediatric patients, those with *RET-NCOA4* tend to have a higher proportion of RAI refractoriness than patients with *RET-CCDC6* (54.5% vs 25%), though *P* = .35 (Table S4 (54)).

**Table 2.** The multiplicative interaction of fusion oncogenes and age group (>20) on radioactive iodine refractoriness

Variants	β	SE	Wald	*P*	OR (95% CI)
Fusions	1.580	0.658	5.761	.016	4.853 (1.336-17.626)
Age group (>20)	2.227	0.555	16.077	<.0001	9.271 (3.122-27.534)
Fusions × age group	−2.273	0.732	9.656	.002	0.103 (0.025-0.432)

### Evaluation of Associations of Major Genetic Alterations With RAI Refractoriness

**Figure 5.**
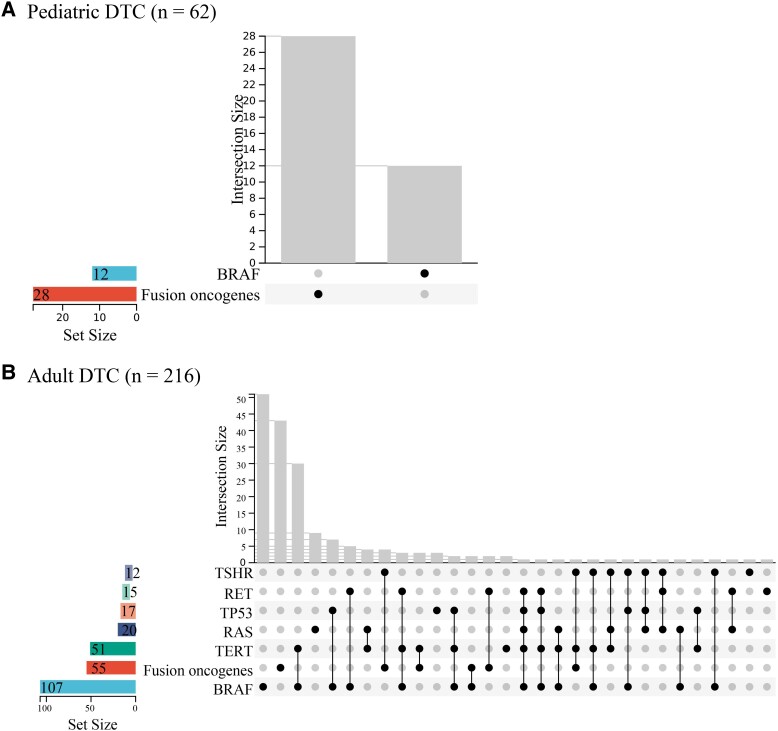
The major genetic alterations in both pediatric and adult patients with locally advanced or distant metastatic DTC. (A) Pediatric cohort. (B) Adult cohort.

**Figure 6.**
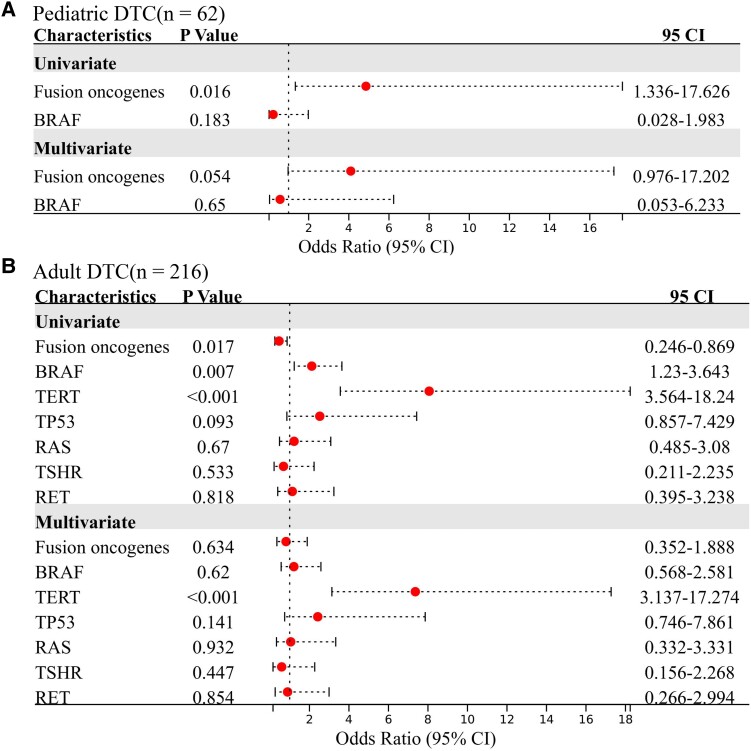
Univariate and multivariate logistic regression analyses were used to evaluate the associations of RAI refractoriness with the major genetic alterations in both pediatric and adult patients with locally advanced or distant metastatic DTC. (A) Pediatric cohort. (B) Adult cohort.

To further explore why the association of fusion oncogenes with RAI refractoriness between different age groups was different, we investigated the major genetic alterations (mutation rate more than 5%) in both pediatric and adult patients. In pediatric patients with DTC, only fusion oncogenes (28/62, 45.2%) and point mutations of *BRAF* (12/62, 19.4%) occurred in more than 5% of patients (Table 3 and Fig. 5A). However, in adult patients with DTC, except for fusion oncogenes (55/216, 25.5%), gene point mutations of *BRAF* (107/216, 49.5%), *TERT* (51/216, 23.6%), *RAS* (20/216, 9.3%), *TP53* (17/216, 7.9%), *RET* (15/216, 6.9%), and *TSHR* (12/216, 5.6%) all occurred in more than 5% of patients (Table 3 and Fig. 5B). Univariate and multivariate logistic regression analyses were performed to investigate the major genetic contributors to RAI refractoriness using data from pediatric and adult patients, respectively. In the pediatric subgroup, only fusion oncogenes appeared to be genetic risk factor of RAI refractoriness, as shown by results from both univariate (*P* = .016, OR 4.85, 95% CI 1.34-17.63) and multivariate (*P* = .054, OR 4.10, 95% CI 0.98-17.20) logistic regression analyses (Fig. 6A). In the adult subgroup, the results from univariate logistic regression analysis demonstrated that both *BRAF* mutation (*P* = .007, OR 2.12, 95% CI 1.23-3.64) and *TERT* mutation (*P* < .001, OR 8.06, 95% CI 3.56-18.24) were significant genetic risk factors for RAI refractoriness while fusion oncogenes were genetic protective factors for RAI refractoriness (*P* = .017, OR 0.46, 95% CI 0.25-0.87) (Fig. 6B). However, the results from multivariate logistic regression analysis in the adult subgroup demonstrated that only *TERT* mutation was a significant risk factor for RAI refractoriness (*P* < .001, OR 7.36, 95% CI 3.14-17.27) (Fig. 6B). The results indicated that fusion oncogenes tended to be the major genetic risk factor for RAI refractoriness in pediatric patients with DTC, while *TERT* mutation was the major genetic risk factor for RAI refractoriness in adult patients with DTC.

**Table 3.** The genetic alterations occurred in more than 5% of pediatric and adult patients with locally advanced or distant metastatic DTC, respectively

	Cases	Mutation ratio (%)	Mutation sites/fusions types
**Pediatric patients**	62		
Fusion oncogenes	28	45.2	*RET-NCOA4 (11), RET-CCDC9 (8), RET-GOLGA5 (1), RET-TRIM27 (1), RET-TRIM33 (1), RET-LMNA (1), ALK-STRN (3), NTRK1-TPR (1), NTRK1-TFG (1)*
*BRAF*	12	19.4	V600E (12)
**Adult patients**	216		
*BRAF^a^*	107	49.5	V600E (106), G606W (2), G596C (2), D594Y (1), G466X (1), G758A (1), L597I (1), W604C (1)
Fusion oncogenes	55	25.5	*RET-NCOA4 (21), RET-CCDC9 (19), RET-ERC1 (5), RET-GOLGA5 (1), RET-PRKG1 (1), RET-TPR (1), RET-TRIM27 (1), NTRK1-TPR (4), NTRK1-SQSTM1 (1), ALK-TRN (1)*
*TERT*	51	23.6	C228T (45), C250T (6)
*RAS^a^*	20	9.3	Q61R (12), Q61K (7), G12C (1), G12V (1), G13D (1)
*TP53^a^*	17	7.9	R337C (2), T125 (2), R156C (2), R196X (2), A161T (1), E285K (1), E339K (1), G245S (1), H179Q (1), R175H (1), R248W (1), R273C (1), R306X (1), S241F (1), V31I (1)
*RET^a^*	15	6.9	R67H (5), R982C (5), V292M (3), R114H (2), A680S (1), A682F (1), G10A (1), L985R (1), N151S (1), P384A (1), Q681E (1), R79Q (1), R287L (1)
*TSHR^a^*	12	5.6	F525S (2), G245S (2), A204V (1), A485D (1), Q704K (1), R450H(1), R528S (1), S305R (1), T399 (1), V689G (1), A623V (1)

Abbreviations: TSHR, thyrotropin receptor.

^*a*^Some patients harbored gene mutations with multiple mutation sites. Fusion oncogenes covered all types of fusions in the above table.

## Discussion

The association between fusion oncogenes and clinicopathological features of DTC has been reported by current literature. For instance, Franco et al (46) have studied the relationship of fusion oncogenes with clinical characteristics in a cohort of 131 pDTCs, which demonstrated that patients with RET/NTRK fusions were more likely to have advanced lymph node and distant metastasis. However, only 56.49% (74/131) of patients in their cohort had locally advanced and/or distant metastasis, which seems insufficiently representative of aggressive and advanced DTCs. Moreover, the results from their study need to be further investigated in aDTC. Herein, we investigated the association between fusion oncogenes and clinical characteristics of locally advanced and/or distant metastatic DTCs in a relatively large cohort of Chinese patients. In our study, both pediatric and adult patients were included in the cohort, thus allowing us to evaluate the implications of fusion oncogenes across age. Our results suggested that fusion oncogenes were significantly associated with increased metastatic capacity in both pediatric and adult DTC. In addition, an interesting tendency between the incidence of fusion oncogenes and age was observed: the younger the patients were, the higher the incidence of fusion oncogenes. Of note, when patients were subgrouped by the age of 20 years, it was found that pDTCs (years <20) had a higher incidence of fusion oncogenes (45.2% vs 25.5%) but a lower incidence of BRAF mutations (19.4% vs 49.5%) than their adult counterparts, which is concordant with previous literature (47, 58, 59).

Moreover, the relationship between fusion oncogenes and RAIR-DTC is another concern. In this study, the associations of fusion oncogenes with RAI refractoriness were investigated in both the pediatric and adult subgroups, and pDTC with fusion oncogenes had a higher possibility of being RAIR-DTC than nonfusion pDTCs. Colombo et al (53) also have investigated the associations of molecular features with RAI refractoriness in a cohort of 70 advanced PTCs, primarily focusing on adult PTCs. Their results suggested both *BRAFV600E* and fusion oncogenes appeared to lead to RAI refractoriness (53). The relevant molecular mechanisms may be related to *NIS*, a crucial mediator of radioiodine uptake, which was found to decrease in pDTC with *RET/NTRK* fusions, which suggested that *RET/NTRK* fusions may affect the uptake of radioiodine in pDTC by downregulating the expression of *NIS* (47). In vitro experiments also demonstrated that *RET* fusions significantly decreased the expression of *PAX8* and *PKA*, led to long-term activation of the *MAPK* pathway, and decreased thyroid differentiation markers such as thyrotropin receptor, *NIS*, and Thyroglobulin (Tg) (60-62). To address the expression pattern of *NIS* in PTC with different genetic alterations, together with the nonavailable samples limited by the retrospective features of our study, public data from GSE151179 was further utilized in this study. The results showed that patients in both the *BRAF* mutation and fusions groups had lower expression of *NIS* than patients in the wild-type group (Fig. S2 (54)). Altogether, these findings suggested that *RET/NTRK* fusions may lead to RAI refractoriness, especially in pDTCs. And they might also shed light on future clinical practice on the redifferentiation of RAI refractoriness by suppressing *RET/NTRK* fusions and increasing the expression of *NIS*.

Interestingly, when aDTCs were separated into a specific subgroup, those with fusion oncogenes (especially *RET/PTC*) appeared to have a lower possibility of being RAIR than those without fusion oncogenes, from which a heterogenic association between fusion oncogenes and RAI refractoriness across the ages seems to be derived. However, when we further compared pDTC and aDTC with fusion oncogenes in terms of RAI refractoriness, no significant difference was observed (pDTC vs aDTC: 39.2% vs 36.3%), while for those without fusion alterations, the proportion of RAI refractoriness in aDTC massively increased (pDTC vs aDTC: 11.7% vs 55.2%). Therefore, we rationally proposed that this so-called “heterogeneity” may be an illusion that was covered by other reasons. Thus, as discussed before, point gene mutations such as *BRAF* and *TERT* were more prevalent in aDTC than fusion oncogenes, and various studies mostly conducted in aDTC have identified that *BRAF* or *TERT* mutations alone or in combination are associated with RAIR-DTC (63, 64). By aberrantly activating the *MAPK* signaling pathway, aPTCs harboring *BRAF* mutations showed a significantly lower thyroid differential score and lower expression of *NIS* than their fusion oncogene-positive counterparts (47, 65, 66). Increasing evidence has demonstrated a strong association between *BRAF* mutations and the loss of RAI avidity in PTC, which could provide a reasonable explanation for the failure of RAI therapy in *BRAF*-mutant PTC (64, 67, 68).

In addition, we further explored the association of fusion oncogenes and those with genetic mutations rates >5% (*BRAF, TERT, TP53, RAS, TSHR*) with the risk of RAI refractoriness. In the pediatric subgroup, only fusion oncogenes and *BRAF* mutations occurred in more than 5% of patients, and it seems that only fusion oncogenes were associated with the risk of RAI refractoriness in the univariate logistic regression analysis. In the adult subgroup, more genetic alterations occurred in more than 5% of patients, including fusion oncogenes, *BRAF, TERT, RAS, TP53, RET,* and *TSHR* mutations, and *TERT* point mutations appeared more associated with RAI refractoriness. In general, patients in the *TERT* mutation group had a higher proportion of RAI refractoriness than those in the Fusions group (84.3% vs 36.3%, *P* < .0001) (Fig. S3 (54)). These findings further supported the heterogenic association of fusion oncogenes with RAI refractoriness between the pediatric and adult subgroups resulting from the different prevalence of point mutations of genes such as *TERT* or *BRAF*. Of note, in our study, we noticed that 75 patients harboring multiple gene mutations (≥2 gene mutations), such as concurrence of *BRAF* mutations and *TERT* mutations, *RAS* mutations and *TERT* mutations, which were reported to be associated with worse prognosis (69-71). Interestingly, *BRAF* mutations and *RAS* mutations have been identified and generally considered mutually exclusive in thyroid cancer (72), but herein we found 3 patients harboring both of them, among which, 2 of 3 patients appeared to be RAIR-DTC. The exact effect of this coexistence on the prognosis still needs to be explored. It is reported that *RAS* mutations may occur later than *BRAF* mutations and correlate with aggressive disease progression of *BRAF*-mutant thyroid cancers (73-75). Moreover, we found that of the 107 *BRAF*-mutant patients in our cohort, 106 patients harbored *BRAF*^V600E^ mutation, the widely accepted common mutation site of *BRAF*, while few additional *BRAF* alterations other than *BRAF*^V600E^ were also identified; although the implication of which remains unknown, the synergistic effect among gene alterations may play a role in the tumor-driven process.

There are several limitations in this study. First, only locally advanced and/or distant metastatic DTCs were involved, with a dearth of early stages; it is also worth repeating with control without any genetic alterations. Second, although a large advanced stage cohort was involved, the sample size of pDTC samples was relatively small, which may possibly lead to a lack of statistical significance in some analyses (eg, the associations of some clinical characteristics with fusion oncogenes). Additionally, the ThyroLead panel applied in this study only evaluated alterations of 26 genes, which may omit some potential genetic alterations related to RAIR-DTC.

In conclusion, fusion oncogenes were more prevalent in pediatric DTCs than in their adult counterparts, and were associated with pediatric RAI refractoriness. However, for adult DTCs, TERT mutation might be the dominant genetic contributor to RAI refractoriness rather than fusion oncogenes.

## Data Availability

Restrictions apply to the availability of some or all data generated or analyzed during this study to preserve patient confidentiality or because they were used under license. The corresponding author will on request detail the restrictions and any conditions under which access to some data may be provided. NGS data in this study can be accessed at https://ngdc.cncb.ac.cn/via accession number HRA004166 from the corresponding author on reasonable request.

## Abbreviations

AJCC: American Joint Committee on Cancer
DTC: differentiated thyroid cancer
NIS: Na(+)/I(–) symporter
PTC: papillary thyroid cancer
RAI: radioactive iodine
RAIR: radioactive iodine refractory

## References

[1] Pizzato M , Li M, Vignat J, et al The epidemiological landscape of thyroid cancer worldwide: GLOBOCAN estimates for incidence and mortality rates in 2020. Lancet Diabetes Endocrinol. 2022;10(4):264‐272. 35271818 10.1016/S2213-8587(22)00035-3

[2] Bostner J , Ahnström Waltersson M, Fornander T, Skoog L, Nordenskjöld B, Stål O. Amplification of CCND1 and PAK1 as predictors of recurrence and tamoxifen resistance in postmenopausal breast cancer. Oncogene. 2007;26(49):6997‐7005. 17486065 10.1038/sj.onc.1210506

[3] Tobin NP , Sims AH, Lundgren KL, Lehn S, Landberg G. Cyclin D1, Id1 and EMT in breast cancer. BMC Cancer. 2011;11(1):417. 21955753 10.1186/1471-2407-11-417 PMC3192789

[4] Zeng H , Chen W, Zheng R, et al Changing cancer survival in China during 2003-15: a pooled analysis of 17 population-based cancer registries. Lancet Glob Health. 2018;6(5):e555‐e567. 29653628 10.1016/S2214-109X(18)30127-X

[5] Haugen BR , Sherman SI. Evolving approaches to patients with advanced differentiated thyroid cancer. Endocr Rev. 2013;34(3):439‐455. 23575762 10.1210/er.2012-1038 PMC3660715

[6] Kebebew E , Clark OH. Locally advanced differentiated thyroid cancer. Surg Oncol. 2003;12(2):91‐99. 12946480 10.1016/s0960-7404(03)00032-x

[7] Wang LY , Nixon IJ, Patel SG, et al Operative management of locally advanced, differentiated thyroid cancer. Surgery. 2016;160(3):738‐746. 27302105 10.1016/j.surg.2016.04.027 PMC5126966

[8] Nixon IJ , Simo R, Newbold K, et al Management of invasive differentiated thyroid cancer. Thyroid. 2016;26(9):1156‐1166. 27480110 10.1089/thy.2016.0064 PMC5118958

[9] Laetitia G , Sven S, Fabrice J. Combinatorial therapies in thyroid cancer: an overview of preclinical and clinical progresses. Cells. 2020;9(4):830. 32235612 10.3390/cells9040830 PMC7226736

[10] Tumino D , Frasca F, Newbold K. Updates on the management of advanced, metastatic, and radioiodine refractory differentiated thyroid cancer. Front Endocrinol (Lausanne). 2017;8:312. 29209273 10.3389/fendo.2017.00312 PMC5702018

[11] Durante C , Haddy N, Baudin E, et al Long-term outcome of 444 patients with distant metastases from papillary and follicular thyroid carcinoma: benefits and limits of radioiodine therapy. J Clin Endocrinol Metab. 2006;91(8):2892‐2899. 16684830 10.1210/jc.2005-2838

[12] Cancer Genome Atlas Research Network . Integrated genomic characterization of papillary thyroid carcinoma. Cell 2014; 159(3):676‐690. 25417114 10.1016/j.cell.2014.09.050 PMC4243044

[13] Yakushina VD , Lerner LV, Lavrov AV. Gene fusions in thyroid cancer. Thyroid. 2018;28(2):158‐167. 29281951 10.1089/thy.2017.0318

[14] Lu Z , Zhang Y, Feng D, Sheng J, Yang W, Liu B. Targeted next generation sequencing identifies somatic mutations and gene fusions in papillary thyroid carcinoma. Oncotarget. 2017;8(28):45784‐45792. 28507274 10.18632/oncotarget.17412 PMC5542227

[15] Zehir A , Benayed R, Shah RH, et al Mutational landscape of metastatic cancer revealed from prospective clinical sequencing of 10,000 patients. Nat Med. 2017;23(6):703‐713. 28481359 10.1038/nm.4333 PMC5461196

[16] Subbiah V , Yang D, Velcheti V, Drilon A, Meric-Bernstam F. State-of-the-art strategies for targeting *RET*-dependent cancers. J Clin Oncol. 2020;38(11):1209‐1221. 32083997 10.1200/JCO.19.02551 PMC7145587

[17] Kohno T , Tabata J, Nakaoku T. REToma: a cancer subtype with a shared driver oncogene. Carcinogenesis. 2020;41(2):123‐129. 31711124 10.1093/carcin/bgz184

[18] Fenton CL , Lukes Y, Nicholson D, Dinauer CA, Francis GL, Tuttle RM. The ret/PTC mutations are common in sporadic papillary thyroid carcinoma of children and young adults. J Clin Endocrinol Metab. 2000;85(3):1170‐1175. 10720057 10.1210/jcem.85.3.6472

[19] Liu R-T , Chou F-F, Wang C-H, et al Low prevalence of RET rearrangements (RET/PTC1, RET/PTC2, RET/PTC3, and ELKS-RET) in sporadic papillary thyroid carcinomas in Taiwan Chinese. Thyroid. 2005;15(4):326‐335. 15876154 10.1089/thy.2005.15.326

[20] Fagin JA , Wells SA. Biologic and clinical perspectives on thyroid cancer. N Engl J Med. 2016;375(11):1054‐1067. 27626519 10.1056/NEJMra1501993 PMC5512163

[21] Grieco M , Santoro M, Berlingieri MT, et al PTC Is a novel rearranged form of the ret proto-oncogene and is frequently detected in vivo in human thyroid papillary carcinomas. Cell. 1990;60(4):557‐563. 2406025 10.1016/0092-8674(90)90659-3

[22] Salvatore D , Santoro M, Schlumberger M. The importance of the RET gene in thyroid cancer and therapeutic implications. Nat Rev Endocrinol. 2021;17(5):296‐306. 33603219 10.1038/s41574-021-00470-9

[23] Romei C , Ciampi R, Elisei R. A comprehensive overview of the role of the RET proto-oncogene in thyroid carcinoma. Nat Rev Endocrinol. 2016;12(4):192‐202. 26868437 10.1038/nrendo.2016.11

[24] Fusco A , Grieco M, Santoro M, et al A new oncogene in human thyroid papillary carcinomas and their lymph-nodal metastases. Nature. 1987;328(6126):170‐172. 3600795 10.1038/328170a0

[25] Stransky N , Cerami E, Schalm S, Kim JL, Lengauer C. The landscape of kinase fusions in cancer. Nat Commun. 2014;5(1):4846. 25204415 10.1038/ncomms5846 PMC4175590

[26] Maeda K , Murakami H, Yoshida R, et al Biochemical and biological responses induced by coupling of Gab1 to phosphatidylinositol 3-kinase in RET-expressing cells. Biochem Biophys Res Commun. 2004;323(1):345‐354. 15351743 10.1016/j.bbrc.2004.08.095

[27] Fukuda T , Kiuchi K, Takahashi M. Novel mechanism of regulation of Rac activity and lamellipodia formation by RET tyrosine kinase. J Biol Chem. 2002;277(21):19114‐19121. 11886862 10.1074/jbc.M200643200

[28] Worby CA , Vega QC, Zhao Y, Chao HH, Seasholtz AF, Dixon JE. Glial cell line-derived neurotrophic factor signals through the RET receptor and activates mitogen-activated protein kinase. J Biol Chem. 1996;271(39):23619‐23622. 8798576 10.1074/jbc.271.39.23619

[29] Trupp M , Scott R, Whittemore SR, Ibáñez CF. Ret-dependent and -independent mechanisms of glial cell line-derived neurotrophic factor signaling in neuronal cells. J Biol Chem. 1999;274(30):20885‐20894. 10409632 10.1074/jbc.274.30.20885

[30] Mitsutake N , Saenko V. Molecular pathogenesis of pediatric thyroid carcinoma. J Radiat Res. 2021;62(Supplement_1):i71‐i77. 33978172 10.1093/jrr/rraa096 PMC8114219

[31] Vaishnavi A , Le AT, Doebele RC. TRKing down an old oncogene in a new era of targeted therapy. Cancer Discov. 2015;5(1):25‐34. 25527197 10.1158/2159-8290.CD-14-0765 PMC4293234

[32] Kelly LM , Barila G, Liu P, et al Identification of the transforming STRN-ALK fusion as a potential therapeutic target in the aggressive forms of thyroid cancer. Proc Natl Acad Sci U S A. 2014;111(11):4233‐4238. 24613930 10.1073/pnas.1321937111 PMC3964116

[33] Chou A , Fraser S, Toon CW, et al A detailed clinicopathologic study of ALK-translocated papillary thyroid carcinoma. Am J Surg Pathol. 2015;39(5):652‐659. 25501013 10.1097/PAS.0000000000000368 PMC4415964

[34] Landa I , Ibrahimpasic T, Boucai L, et al Genomic and transcriptomic hallmarks of poorly differentiated and anaplastic thyroid cancers. J Clin Invest. 2016;126(3):1052‐1066. 26878173 10.1172/JCI85271 PMC4767360

[35] Bastos AU , de Jesus AC, Cerutti JM. ETV6-NTRK3 And STRN-ALK kinase fusions are recurrent events in papillary thyroid cancer of adult population. Eur J Endocrinol. 2018;178(1):83‐91. 29046324 10.1530/EJE-17-0499

[36] Bauer AJ . Molecular genetics of thyroid cancer in children and adolescents. Endocrinol Metab Clin North Am. 2017;46(2):389‐403. 28476228 10.1016/j.ecl.2017.01.014

[37] Cherella CE , Wassner AJ. Pediatric thyroid cancer: recent developments. Best Pract Res Clin Endocrinol Metab. 2022;37(1):101715. 36404191 10.1016/j.beem.2022.101715

[38] Paulson VA , Rudzinski ER, Hawkins DS. Thyroid cancer in the pediatric population. Genes (Basel). 2019;10(9):723. 31540418 10.3390/genes10090723 PMC6771006

[39] Gertz RJ , Nikiforov Y, Rehrauer W, McDaniel L, Lloyd RV. Mutation in BRAF and other members of the MAPK pathway in papillary thyroid carcinoma in the pediatric population. Arch Pathol Lab Med. 2016;140(2):134‐139. 26910217 10.5858/arpa.2014-0612-OA PMC8006595

[40] Fukahori M , Yoshida A, Hayashi H, et al The associations between RAS mutations and clinical characteristics in follicular thyroid tumors: new insights from a single center and a large patient cohort. Thyroid. 2012;22(7):683‐689. 22650231 10.1089/thy.2011.0261

[41] Garcia-Rostan G , Zhao H, Camp RL, et al *Ras* mutations are associated with aggressive tumor phenotypes and poor prognosis in thyroid cancer. J Clin Oncol. 2003;21(17):3226‐3235. 12947056 10.1200/JCO.2003.10.130

[42] Xing M . Molecular pathogenesis and mechanisms of thyroid cancer. Nat Rev Cancer. 2013;13(3):184‐199. 23429735 10.1038/nrc3431 PMC3791171

[43] Sobrinho-Simões M , Máximo V, Rocha AS, et al Intragenic mutations in thyroid cancer. Endocrinol Metab Clin North Am. 2008;37(2):333‐362. 18502330 10.1016/j.ecl.2008.02.004

[44] Penko K , Livezey J, Fenton C, et al BRAF Mutations are uncommon in papillary thyroid cancer of young patients. Thyroid. 2005;15(4):320‐325. 15876153 10.1089/thy.2005.15.320

[45] Kumagai A , Namba H, Saenko VA, et al Low frequency of BRAFT1796A mutations in childhood thyroid carcinomas. J Clin Endocrinol Metab. 2004;89(9):4280‐4284. 15356022 10.1210/jc.2004-0172

[46] Franco AT , Ricarte-Filho JC, Isaza A, et al Fusion oncogenes are associated with increased metastatic capacity and persistent disease in pediatric thyroid cancers. J Clin Oncol. 2022;40(10):1081‐1090. 35015563 10.1200/JCO.21.01861 PMC8966969

[47] Lee YA , Lee H, Im S-W, et al NTRK And RET fusion-directed therapy in pediatric thyroid cancer yields a tumor response and radioiodine uptake. J Clin Invest. 2021;131(18):e144847. 34237031 10.1172/JCI144847 PMC8439610

[48] Liu M , Chen P, Hu H-Y, et al Kinase gene fusions: roles and therapeutic value in progressive and refractory papillary thyroid cancer. J Cancer Res Clin Oncol. 2021;147(2):323‐337. 33387037 10.1007/s00432-020-03491-5 PMC11801884

[49] Perri F , Pezzullo L, Chiofalo MG, et al Targeted therapy: a new hope for thyroid carcinomas. Crit Rev Oncol Hematol. 2015;94(1):55‐63. 25465739 10.1016/j.critrevonc.2014.10.012

[50] Vallejo Casas JA , Sambo M, López López C, et al Initial clinical and treatment patterns of advanced differentiated thyroid cancer: ERUDIT study. Eur Thyroid J. 2022;11(5):e210111. 35900793 10.1530/ETJ-21-0111 PMC9422238

[51] Naoum GE , Morkos M, Kim B, Arafat W. Novel targeted therapies and immunotherapy for advanced thyroid cancers. Mol Cancer. 2018;17(1):51. 29455653 10.1186/s12943-018-0786-0 PMC5817719

[52] Haugen BR , Alexander EK, Bible KC, et al American Thyroid Association management guidelines for adult patients with thyroid nodules and differentiated thyroid cancer: the American Thyroid Association guidelines task force on thyroid nodules and differentiated thyroid cancer. Thyroid. 2015;2016(1):26. 10.1089/thy.2015.0020 PMC4739132 26462967

[53] Colombo C , Minna E, Gargiuli C, et al The molecular and gene/miRNA expression profiles of radioiodine resistant papillary thyroid cancer. J Exp Clin Cancer Res. 2020;39(1):245. 33198784 10.1186/s13046-020-01757-x PMC7667839

[54] Gaoda Ju YS , Wang H, Zhang X, et al Supplemental material for “Fusion oncogenes in patients with locally advanced or distant metastatic differentiated thyroid cancer”. Zenodo Repository. (2023)

[55] Haugen BR . 2015 American Thyroid Association management guidelines for adult patients with thyroid nodules and differentiated thyroid cancer: what is new and what has changed? Cancer. 2017;123(3):372‐381. 27741354 10.1002/cncr.30360

[56] Tan L-C , Liu W-L, Zhu X-L, et al Next-generation sequencing enhances the diagnosis efficiency in thyroid nodules. Front Oncol. 2021;11:677892. 34322384 10.3389/fonc.2021.677892 PMC8312558

[57] Gudmundsson J , Sulem P, Gudbjartsson DF, et al A study based on whole-genome sequencing yields a rare variant at 8q24 associated with prostate cancer. Nat Genet. 2012;44(12):1326‐1329. 23104005 10.1038/ng.2437 PMC3562711

[58] Zanella AB , Scheffel RS, Weinert L, Dora JM, Maia AL. New insights into the management of differentiated thyroid carcinoma in children and adolescents (review). Int J Oncol. 2021;58(5):13. 33649842 10.3892/ijo.2021.5193

[59] Fagin JA . Age of onset of receptor tyrosine kinase fusions drives distinct biologic outcomes in thyroid cancer. J Clin Oncol. 2022;40(10):1124‐1126. 35104153 10.1200/JCO.21.02864

[60] Wang J , Knauf JA, Basu S, et al Conditional expression of RET/PTC induces a weak oncogenic drive in thyroid PCCL3 cells and inhibits thyrotropin action at multiple levels. Mol Endocrinol. 2003;17(7):1425‐1436. 12690093 10.1210/me.2003-0041

[61] Venkateswaran A , Marsee DK, Green SH, Jhiang SM. Forskolin, 8-Br-3′,5′-cyclic adenosine 5′-monophosphate, and catalytic protein kinase A expression in the nucleus increase radioiodide uptake and sodium/iodide symporter protein levels in RET/PTC1-expressing cells. J Clin Endocrinol Metab. 2004;89(12):6168‐6172. 15579773 10.1210/jc.2004-1414

[62] Trapasso F , Iuliano R, Chiefari E, et al Iodide symporter gene expression in normal and transformed rat thyroid cells. Eur J Endocrinol. 1999;140(5):447‐451. 10229912 10.1530/eje.0.1400447

[63] Yang X , Li J, Li X, et al TERT promoter mutation predicts radioiodine-refractory character in distant metastatic differentiated thyroid cancer. J Nucl Med. 2017;58(2):258‐265. 27493271 10.2967/jnumed.116.180240

[64] Yang K , Wang H, Liang Z, Liang J, Li F, Lin Y. BRAFV600E mutation associated with non-radioiodine-avid status in distant metastatic papillary thyroid carcinoma. Clin Nucl Med. 2014;39(8):675‐679. 24978326 10.1097/RLU.0000000000000498

[65] Xing M . BRAF Mutation in papillary thyroid cancer: pathogenic role, molecular bases, and clinical implications. Endocr Rev. 2007;28(7):742‐762. 17940185 10.1210/er.2007-0007

[66] Riesco-Eizaguirre G , Gutiérrez-Martínez P, García-Cabezas MA, Nistal M, Santisteban P. The oncogene BRAF V600E is associated with a high risk of recurrence and less differentiated papillary thyroid carcinoma due to the impairment of na+/I- targeting to the membrane. Endocr Relat Cancer. 2006;13(1):257‐269. 16601293 10.1677/erc.1.01119

[67] Xing M , Westra WH, Tufano RP, et al BRAF Mutation predicts a poorer clinical prognosis for papillary thyroid cancer. J Clin Endocrinol Metab. 2005;90(12):6373‐6379. 16174717 10.1210/jc.2005-0987

[68] Ricarte-Filho JC , Ryder M, Chitale DA, et al Mutational profile of advanced primary and metastatic radioactive iodine-refractory thyroid cancers reveals distinct pathogenetic roles for BRAF, PIK3CA, and AKT1. Cancer Res. 2009;69(11):4885‐4893. 19487299 10.1158/0008-5472.CAN-09-0727 PMC2690720

[69] Cao J , Zhu X, Sun Y, Li X, Yun C, Zhang W. The genetic duet of BRAF V600E and TERT promoter mutations predicts the poor curative effect of radioiodine therapy in papillary thyroid cancer. Eur J Nucl Med Mol Imaging. 2022;49(10):3470‐3481. 35501518 10.1007/s00259-022-05820-x

[70] Pappa T , Ahmadi S, Marqusee E, et al Oncogenic mutations in PI3K/AKT/mTOR pathway effectors associate with worse prognosis in BRAFV600E -driven papillary thyroid cancer patients. Clin Cancer Res. 2021;27(15):4256‐4264. 34088725 10.1158/1078-0432.CCR-21-0874

[71] Bikas A , Ahmadi S, Pappa T, et al Additional oncogenic alterations in RAS-driven differentiated thyroid cancers associate with worse clinicopathologic outcomes. Clin Cancer Res. 2023;29(14):2678‐2685. 37260297 10.1158/1078-0432.CCR-23-0278 PMC10524472

[72] Kimura ET , Nikiforova MN, Zhu Z, Knauf JA, Nikiforov YE, Fagin JA. High prevalence of BRAF mutations in thyroid cancer: genetic evidence for constitutive activation of the RET/PTC-RAS-BRAF signaling pathway in papillary thyroid carcinoma. Cancer Res. 2003;63(7):1454‐1457. 12670889

[73] Zou M , Baitei EY, Alzahrani AS, et al Concomitant RAS, RET/PTC, or BRAF mutations in advanced stage of papillary thyroid carcinoma. Thyroid. 2014;24(8):1256‐1266. 24798740 10.1089/thy.2013.0610 PMC4106383

[74] Costa AM , Herrero A, Fresno MF, et al BRAF Mutation associated with other genetic events identifies a subset of aggressive papillary thyroid carcinoma. Clin Endocrinol (Oxf). 2008;68(4):618‐634. 18070147 10.1111/j.1365-2265.2007.03077.x

[75] Cabanillas ME , Dadu R, Iyer P, et al Acquired secondary RAS mutation in BRAFV600E-mutated thyroid cancer patients treated with BRAF inhibitors. Thyroid. 2020;30(9):1288‐1296. 32216548 10.1089/thy.2019.0514 PMC7869871

